# *FOXP2* contributes to the cognitive impairment in chronic patients with schizophrenia

**DOI:** 10.18632/aging.102198

**Published:** 2019-08-19

**Authors:** Xiaoe Lang, Wenzhong Zhang, Xinxin Song, Guangya Zhang, Xiangdong Du, Yongjie Zhou, Zezhi Li, Xiang Yang Zhang

**Affiliations:** 1 Department of Psychiatry, The First Clinical Medical College, Shanxi Medical University, Taiyuan, China; 2 Qingdao Mental Health Center, Qingdao, China; 3 Suzhou Psychiatric Hospital, The Affiliated Guangji Hospital of Soochow University, Suzhou, China; 4 Research Center for Psychological and Health Sciences, China University of Geosciences, Wuhan, China; 5 Affiliated Wuhan Mental Health Center, Tongji Medical College of Huazhong University of Science and Technology, Wuhan, China; 6 Department of Neurology, Ren Ji Hospital, Shanghai Jiao Tong University School of Medicine, Shanghai, China; 7 Department of Psychiatry and Behavioral Sciences, The University of Texas Health Science Center at Houston, Houston, TX 77054, USA

**Keywords:** schizophrenia, cognition, FOXP2, polymorphism

## Abstract

The forkhead-box P2 (*FOXP2*), involving in language and memory function, has been identified as susceptibility to schizophrenia. However, no study examined the role of *FOXP2* on cognitive impairment in schizophrenia. Total 1106 inpatients with schizophrenia and 404 controls were recruited and genotyped. Among them, 867 patients and 402 controls were assessed through the Repeatable Battery for the Assessment of Neuropsychological Status (RBANS). SHEsis software was used to investigate the association of *FOXP2* rs10447760 with schizophrenia, followed by logistic regression. The model of covariance (ANCOVA) and multivariate analysis were conducted to investigate the effect of *FOXP2* rs10447760 on cognitive impairment in schizophrenia. No differences in the genotypic and allelic frequencies of the *FOXP2* rs10447760 were found between patients and controls (both p> 0.05). Except for the visuospatial/constructional score (p > 0.05), other five RBANS scores were lower in patients compared to controls (all p < 0.0001). Interestingly, we found immediate memory score was lower in patients carrying genotype CT compared to genotype CC (F=5.19, p=0.02), adjusting for confounding data. Our study suggested that *FOXP2* rs10447760 has no effect on the susceptibility to schizophrenia, while it may be associated with its cognitive impairment, especially immediate memory in chronic schizophrenia.

## INTRODUCTION

Schizophrenia is a common mental disorder causing high disability and heavy economic burden worldwide, characterized by cardinal features including psychotic symptoms and cognitive impairments [1]. Schizophrenia was called “dementia praecox” historically for the existed cognitive impairments in memory, attention, visuospatial, language and learning [2, 3]. Previous lines of evidence also demonstrated that either first-onset or chronic patients with schizophrenia displayed impairments of cognitive domains involving in attention, memory, learning, and executive functioning [4, 5]. Cognitive impairment was usually present in the prodromal phase of schizophrenia and persisted throughout the duration of the disease [6, 7]. Furthermore, cognitive impairment in schizophrenia can predict the functional outcomes in patients, affect quality of life, employment status and social function, and even impeded treatment and rehabilitation [8, 9]. Therefore, cognitive recovery is considered to be the main target of clinical treatment for schizophrenia [10]. However, until now, the underlying pathophysiological mechanisms of cognitive impairment in schizophrenia remain obscure.

Previous family and twins studies demonstrated that genetic aspects explained the variability in cognition including 50% in memory, 70% in verbal reasoning, and 79% in abstract reasoning [11–13]. Furthermore, cognitive impairment was also present in unaffected relatives of schizophrenia [14, 15]. These evidences indicated genetic factors may contribute to cognitive impairment of schizophrenia. Recently, neurodevelopmental hypothesis of schizophrenia has generally been accepted, at least partly, based on cognitive impairment presenting in schizophrenia [16, 17]. Thus, neurodevelopmental dysfunction may also be reasonable to be involved in mechanisms of cognitive impairment in schizophrenia.

The forkhead-box P2 (*FOXP2*) is involved in the development and function of the brain. *FOXP2* is located on chromosome 7q31 which has been identified as the susceptible locus for schizophrenia through genome-wide association studies [18]. *FOXP2* was previously reported as a causative gene for language and speech disorders, especially in a large three-generation family [19]. Furthermore, there was evidence that FOXP*2* contributed to the development of the neuron which influenced language and speech function [20, 21]. A previous study determined that *FOXP2* mRNA was most expressed in brain deep cortical neurons, striatum medium spiny neurons, and cerebellar Purkinje cells in mouse and human development, and these neuronal cells were essential for language function [22].

As we all know, speech and language functions are essential components of cognition. Previous studies indicated that specific language-related circuits were also impaired in both patients with schizophrenia and in those relatives with high genetic risk for developing schizophrenia [23, 24]. Tolosa et al. [25] found significant association of *FOXP2* polymorphism rs2253478 with poverty of speech in schizophrenia. These findings suggest that *FOXP2* may play a crucial role in pathophysiology of cognitive impairment in schizophrenia. Recently, the *FOXP2* polymorphism rs10447760, located in the 5′regulatory region was found to correlated with schizophrenia. For example, Sanjuán et al. [26] demonstrated that the haplotype of *FOXP2*, containing polymorphism rs10447760, contributed to the vulnerability to schizophrenia with auditory hallucinations. Recently, Li et al. [27] also showed that *FOXP2* polymorphism rs10447760 correlated with the suspectability of schizophrenia in Han population. However, some studies did not replicate these results. For example, the recent studies showed no correlation of *FOXP2* polymorphism rs10447760 with schizophrenia in Han population [28, 29]. Interestingly, the most recent meta-analysis demonstrated *FOXP2* rs10447760 conferred the vulnerability to schizophrenia in Caucasians, however, such association was not found in Chinese Han population [30]. Furthermore, our recent study demonstrated significant correlation between *FOXP2* rs10447760 and the symptoms of chronic patients with schizophrenia in a Chinese Han population [28].

From the results mentioned above, we hypothesized that *FOXP2* polymorphism rs10447760 might contribute to the cognitive impairment, especially the language function in patients with schizophrenia. To our best knowledge, there has been no study to investigate the association of cognitive performance with *FOXP2* polymorphisms in schizophrenia. Therefore, the main aim of present study was to determine the effect of this polymorphism on cognitive impairment in schizophrenia.

## RESULTS

### Association analysis between FOXP2 rs10447760 and schizophrenia

A total of 1106 patients and 404 healthy controls who completed *FOXP2* rs10447760 genotyping were enrolled (Table 1). Except for age, there were significant differences in sex, years of education, and BMI between them (all p<0.01), and they were controlled as covarites in the following statistical analyses.

**Table 1:** Demographic characteristics in patients with schizophrenia and healthy controls (Mean ± SD).

**Variable**	**Association between rs10447760 and schizophrenia**				**Association between rs10447760 and cognition score**			
	**Patients (N=1106)**	**Controls (N=404)**	**Statistic**	***p***	**Patients (N=867)**	**Controls (N=402)**	**Statistic**	***p***
Age (Years)	45.93±10.79	44.70±13.50	1.64	0.10	45.48±10.71	44.70±13.50	1.01	0.31
Gender (Male)	831 (75.1%)	162 (40.1%)	161.33	<0.0001	684 (78.9%)	158 (39.3%)	191.50	<0.0001
Education (Years)	8.73±4.76	9.71±5.59	3.21	0.001	8.88±5.02	9.71±5.59	2.61	0.009
BMI	23.94±4.09	25.14±4.26	4.71	<0.0001	23.99±4.16	25.02±4.61	3.73	<0.0001
Age of onset	25.42±8.65	-	-	-	24.72±7.20	-	-	-
Duration of illness(Years)	24.17±9.34	-	-	-	23.82±9.70	-	-	-
Atypical antipsychotic (N)	779	-	-	-	604	-	-	-
Typical antipsychotic(N)	327	-	-	-	263	-	-	-
Daily antipsychotic dose(chlorpromazine equivalents), mg/d	396.90±390.96	-	-	-	394.06±322.09	-	-	-
Duration of current antipsychotic treatment (Months)	31.93±37.63	-	-	-	31.28±44.08	-	-	-
PANSS score	
Positive symptoms	12.44±5.94	-	-	-	11.29±5.04	-	-	-
Negative symptoms	20.86±8.98	-	-	-	19.89±8.29	-	-	-
General psychopathology	25.98±7.25	-	-	-	23.72±3.82	-	-	-
Total score	59.29±16.56	-	-	-	54.90±11.78	-	-	-

^a^Total 1106 patients and 404 healthy controls; ^b^Total 867 patients and 402 healthy controls who had completed both genotyping and cognitive assessment.

SHEsis analysis demonstrated that the distributions of *FOXP2* rs10447760 genotype both in patient group and control group were consistent with Hardy-Weinberg equilibrium (case: χ^2^ =0.25, p=0.61; control: χ^2^ =0.02, p is 0.90). No significant differences were found in the genotype and allele distributions of *FOXP2* rs10447760 between patients and controls (χ^2^= 3.67, P = 0.06; χ^2^ = 3.63, P = 0.06; respectively). Concerning that the sex, years of education and BMI differed between patients and controls, logistic regression analysis (Backward conditional) was conducted to control for these confounding factors. There were still no differences in the *FOXP2* rs10447760 genotype and allele distributions between patient and control groups (both p>0.05).

### Cognitive score between patients with schizophrenia and healthy controls

867 patients and 402 controls had completed both genotyping and cognitive assessment (Table 1). The patients included 684 males and 183 females, with mean age of 45.48±10.71 years, the duration of illness of 23.82±9.7 years, and the average educational levels of 8.88±5.2 years.

Except for age, there were significant differences in sex, education, and BMI between the patient group and the control group (all p<0.01), which were controlled for in the following covariance (ANCOVA) models. Except the visuospatial/constructional score (p > 0.05), ANCOVA showed that other RBANS scores were significantly lower in patient group than those in control group (all p < 0.0001) after adjusting for confounding factors including sex, education and BMI.

### Association of the FOXP2 rs10447760 with cognitive score

Further ANCOVA showed that immediate memory score was significantly lower only in patients with genotype CT than those in patients with genotype CC (F=5.19, p=0.02), after controlling for age, sex, years of education, illness duration, age of onset, antipsychotic types, antipsychotic daily dose and current antipsychotic medication duration (Table 2). However, no effects of genotype and genotype X diagnosis were found on any RBANS scores (all p > 0.05) (Table 2).

**Table 2:** Comparisons among the RBANS total and five subscale scores by diagnostic and genotypic groupings (Mean ± SD).

**RBANS scores**	**Patients with schizophrenia**		**Controls**		**Diagnosis**		**Genotype**		**Diagnosis × Genotype**	
	**CC(N=836)**	**CT(N=31)**	**CC(N=397)**	**CT(N=5)**	**F**	**p**	**F**	**p**	**F**	**p**
Immediate memory	57.77±16.31^a^	51.29±13.43^a^	75.64±17.28	78.60±23.52	28.73	<0.0001	0.47	0.49	0.85	0.36
Attention	64.31±18.57	61.03±13.98	87.51±20.31	80.20±20.63	20.97	<0.0001	1.31	0.25	0.19	0.66
Language	74.77±18.11	71.94±18.10	94.02±13.03	86.00±17.94	16.90	<0.0001	1.79	0.18	0.41	0.52
Visuospatial/ construction	76.67±18.19	75.16±17.49	79.75±15.52	71.80±18.74	0.001	0.97	1.26	0.26	0.58	0.45
Delayed memory	64.23±19.54	62.19±19.08	86.21±15.25	87.80±17.68	28.69	<0.0001	0.003	0.96	0.17	0.68
Total score	61.14±14.55	57.94±10.72	79.88±15.57	76.00±19.85	26.13	<0.0001	0.97	0.33	0.01	0.93

^a^ A significant genotypic effect on the immediate memory in patients with schizophrenia: immediate memory score was significantly lower in patients with CT genotype than those with CC genotype, *p*= 0.02.

Multivariate regression (stepwise) further demonstrated the following variables independently correlated with the immediate memory score in patient group: the *FOXP2* rs10447760 genotype (β=-7.01, t=2.39, p=0.02), age (β=-0.28, t=4.64, p < 0.001), years of education (β=0.51, t=3.06, p = 0.002), and illness duration (β=0.03, t=3.35, p =0.001).

This sample had 0.83~0.92 statistical power to examine the association of this polymorphism with schizophrenia with log additive, and the assumption of a moderate size effect of 1.5 (α< 0.05, two-tailed test).

## DISCUSSION

To our knowledge, the present study firstly examines the association of *FOXP2* rs10447760 with cognitive impairment in schizophrenia. The main results were as follow (1) *FOXP2* rs10447760 may not be associated with suspectability of schizophrenia; (2) *FOXP2* rs10447760 correlated with immediate memory only in patients with schizophrenia, showing that immediate memory score was lower in patients with genotype CT than those in patients with genotype CC.

A recent study identified *FOXP2* as susceptible loci for schizophrenia using genome-wide association method [18]. The same research group demonstrated that *FOXP2* polymorphism rs10447760 was strongly correlated with susceptibility of schizophrenia in Chinese Han population [27]. However, this finding was not confirmed in some other recent studies including our current study in Chinese Han population [28, 29]. The inconsistent results also occurred in the studies among the Caucasians. For example, Sanjuán et al. found that the haplotype including rs10447760 was associated with schizophrenia [26], while Tolosa et al. did not confirm this association [25]. The most recent meta-analysis indicated that *FOXP2* rs10447760 significantly correlated with susceptibility schizophrenia in Caucasians, but not in Chinese Han population [30]. The main reason for the different results in Chinese Han population may result from the rare variant of rs10447760 in the Chinese Han population. We could not detect TT genotype in the subjects in our current study, and only 1.2% of healthy controls had CT genotype, which is consistent with three recent association studies [27–29]. It is worthy of noting that rare variants can easily lead to statistical variation. Even one more or less low-frequency genotype could change the significance of the p value.

Interestingly, our results showed significant association between *FOXP2* rs10447760 and cognitive performance in schizophrenia. We found that *FOXP2* rs10447760 was correlated with immediate memory, showing poor immediate memory score in patients carrying CT genotype than that in patients carrying CC genotype. However, we did not find that *FOXP2* rs10447760 was correlated with language function in schizophrenia. Some studies have demonstrated *FOXP2* was associated with some cognitive performance, such as memory. For example, FOXP2-expressing spinal neurons could adjust song motor output to tutor song memory during learning [31]. Recent study showed that affected KE members (aKE) (patients with *FOXP2* gene mutation) selectively impaired the phonological working memory [32]. Schreiweis et al. [33] demonstrated that humanized FOXP2 promoted learning performance by heightening transitions from declarative to procedural function, suggesting that FOXP2 had a striking specific effect on learning dynamics, striatal dopamine levels, and synaptic plasticity. These studies provided the evidence that *FOXP2* gene could regulate memory function through being expressed in specific regions of the brain. However, we found no effects of *FOXP2* rs10447760 on language function in schizophrenia which was consistent with recent study [34].

Although no direct evidence shows that *FOXP2* rs10447760 is a functional SNP, we found that it was correlated with immediate memory. Previous study demonstrated that this SNP rs10447760 could regulate the FOXP2 expression in thalamus and white matter area of the brain by using eQTL analysis extracted from the BRAINEAC database [30].

Furthermore, previous studies have shown that *FOXP2* mRNAs were highly preferentially expressed in the striatum during neuronal development, and it also remained in striosomes in the mature striatum [22, 35]. Such preferential expression is unique, since *FOXP2* is preferentially expressed in the “shell” domain in ventral striatum, which was found to connect with limbic function [22, 35]. The striatum is well known to contribute to the procedure of programmed memory, memory function [36, 37] and the formation of a memory network connected with the hippocampus [38]. White matter has also been proven to be correlated with clinical symptoms, cognitive deficiency and social cognition in patients with schizophrenia [39–41]. Taken together, we speculated that *FOXP2* rs10447760 might affect *FOXP2* expression in specific region of the brain to regulate memory function. However, the precise molecular mechanisms underlying the association between *FOXP2* rs10447760 and immediate memory warrant further investigation.

Some limitations should be concerned in our present study. Firstly, as noted above, *FOXP2* rs10447760 has the rare variant allele T and we even did not detect TT genotype. Thus, a small number of patients with CT genotype could have changed the significance of p value dramatically, and we could not rule out the false positive results. Secondly, patients enrolled in present study were treated with different antipsychotics for a long-term period, which could affect cognitive function in chronic patients with schizophrenia. Thirdly, we just examined a single genetic polymorphism effect on cognitive impairments of patients with schizophrenia, and other polymorphisms and genes interaction should be concerned [42]. Last but not the least, the RBANS has five domains including immediate memory, attention, language, visuospatial/ constructional, and delayed memory. However, in fact, there are more cognitive domains such as executive function, working memory [43], emotional management, and facial emotion perception [44–45]. In the future study, more cognitive domains should be examined and the different ethnic population should be recruited to confirm our findings.

In summary, we found that *FOXP2* polymorphism rs10447760 may not be involved in the susceptibility to schizophrenia, but may contribute to cognitive performance, especially immediate memory in schizophrenia. Patients carrying CT genotype had the greater cognitive impairment in immediate memory than those with CC genotype. Thus, *FOXP2* rs10447760 may be involved in pathophysiological mechanisms for cognitive impairments in schizophrenia. Moreover, the chronic patients with schizophrenia displayed extensive severe cognitive impairments shown on all of RBANS scores compared to healthy controls. However, our findings still warrant to be confirmed in larger samples from different ethnics before the firm conclusion could be made.

## MATERIALS AND METHODS

### Subjects

The protocol was reviewed and approved by Institutional Review Board of Beijing Hui-Long-Guan hospital. Written informed consents were obtained from all participants. All subjects gave written informed consent in accordance with the Declaration of Helsinki. The patients were enrolled from the inpatients, meeting the inclusion criteria as following: (1) Han Chinese patients with age from 18 to 75 years; (2) satisfying the diagnosis of schizophrenia by the Diagnostic and Statistical Manual of Mental Disorders, Fourth Edition (DSM-IV) according to the screening of Structure Clinical Interview for DSM-IV (SCID- I/P) by two psychiatrists; (3) with at least 5-year illness duration; (4) with stable doses of oral antipsychotics for at least 12 months.

A total of 1106 patients were enrolled, and among them, 867 patients completed cognition assessment (mean age of 45.48±10.71 years, 684 males and 183 females), with the duration of illness of 23.82±9.70 years, and the average education levels of 8.88±5.2 years (demographic data and clinical characteristics were shown in Table 1). They had received current antipsychotic treatment for 31.28±44.08 months, and had mainly been treated with one antipsychotic drug including clozapine (n = 370), risperidone (n = 164), quetiapine (n = 70), chlorpromazine (n = 55), sulpiride (n = 38), aripiprazole (n = 35), perphenazine (n = 27), olanzapine (n = 23), haloperidol (n = 16), and other antipsychotics (n = 69). The doses of antipsychotics equivalent to chlorpromazine were 394.06±322.09 mg/day [46–48].

A total 404 healthy controls were enrolled through advertisement. Individuals with family history of mental disorders or any major Axis I disorders were excluded.

Any subjects with cardiovascular disease, cerebrovascular disease, infections, cancer, unstable diabetes, uncontrolled hypertension, and pregnancy were excluded. Any subjects with drug or alcohol abuse/dependence determined by the laboratory urine tests were excluded.

### Clinical and cognitive assessments

The self-designed questionnaire including socio-demographic profile, physical and psychological situation was collected from all the subjects by the interview of researchers. The Repeatable Battery for the Assessment of Neuropsychological Status (RBANS, Form A) [49] was used to assess the patient’s cognitive function. The RBANS has been translated into Chinese version and the clinical validity and test - retest reliability of the Chinese version of RBANS was established [28]. The RBANS contains 12 subtests, and five age-adjusted index scores were calculated with a total score. The five indexes scores include immediate memory, attention, language, visuospatial/ constructional, and delayed memory. All the patients were measured with the RBANS in their stable state when they did not present acute psychotic symptoms or deterioration of function.

### DNA isolation and SNP genotyping

A total of 5 ml peripheral whole blood samples were abstracted in tube with anticoagulant ethylene diamine tetraacetic acid (EDTA), and genomic DNA was extracted from whole blood samples using a salting- out method [28]. The *FOXP2* polymorphism rs10447760 was genotyped by using Matrix-Assisted Laser Desorption/Ionization Time of Flight Mass Spectrometry (MALDI-TOF MS) (Sequenom Inc., San Diego, CA, USA) according to the protocol [28]. The primers and extent sequence were determined according to the NCBI GenBank database (Sense: 5′- ACGTTGGATGA ACACTGC-AGGCTTTGTTCG-3′, Antisense: 5′- ACGT TGGATGTTTGGAGTCAGCTAGCAC-AG-3′) (extent sequence: CAGAGCGCTAAACCC). Genotyping process was conducted by researchers who were blind to the clinical information. Regarding quality control and verification, 5% samples were randomly duplicate with an error rate of <0.1%.

### Statistical analyses

T-test for continuous variables and chi-square (χ2) test for categorical variables were used as appropriate. Hardy - Weinberg equilibrium, genotypic and allelic frequencies of *FOXP2* rs10447760 were evaluated through SHEsis (http://analysis.bio-x.cn) [50]. Further, to determine the effect of *FOXP2* rs10447760 on suspectibility of schizophrenia, logistic regression was used to control the confounding factors.

The models of covariance (ANCOVA) were based on five subscale and total scores of RBANS as the dependent variables respectively, with the diagnosis and the *FOXP2* rs10447760 genotype as the independent variables, with sex, BMI, and years of education as the covariates. Also, the main effects of diagnosis, genotype, and genotype × diagnosis in each model were examined. Further, ANCOVA was used to determine the differences in RBANS scores in accordance with the genotypic groups in patient group or control group respectively, with sex, education and BMI as covariates in control group, together with duration of illness, age of onset, antipsychotic types, antipsychotic daily dose, and current antipsychotics medication duration as covariates in patient group. Bonferroni correction was performed to adjust for each multiple test. Further, we used multivariate analysis (stepwise) with the positive results as the dependent variables to examine the effects of variables including the *FOXP2* rs10447760 genotype, age, sex, education, duration of illness, age of onset, antipsychotic types, antipsychotic daily dose and current antipsychotic medication duration in patients.

Power analysis was conducted using software Quanto (Version 1.2.3). All statistical analysis were conducted by the PASW Statistics 18.0 software (SPSS Inc., Chicago, IL, USA). All p values were two-tailed at the significant level of below 0.05.

### Ethical statement

The protocol was reviewed and approved by Institutional Review Board of Beijing Hui-Long-Guan hospital. Written informed consents were obtained from all participants. All subjects gave written informed consent in accordance with the Declaration of Helsinki.
